# PAE Coding System and Ethogram of Wild Yunnan Snub-Nosed Monkeys with Behavioral Comparisons Based on Infrared Camera Data

**DOI:** 10.3390/biology15171509

**Published:** 2026-09-03

**Authors:** Hanyu Zhu, Duji Zhaba, Hong Xie, Dayong Li, Wancai Xia

**Affiliations:** 1 Key Laboratory of Southwest China Wildlife Resources Conservation (Ministry of Education), China West Normal University, Nanchong 637009, China; 18084802573@163.com (H.Z.); 18282086950@163.com (H.X.); 2 Yunnan Baimaxueshan National Nature Reserve, Diqing 674400, China; 13508876532@163.com

**Keywords:** *Rhinopithecus bieti*, infrared camera, PAE coding system, ethogram, behavioral difference comparison

## Abstract

The standardized construction of ethograms and the quantitative comparison of behavioral differences are fundamental to animal behavioral ecology research. This study focused on the Yunnan snub-nosed monkey (*Rhinopithecus bieti*) inhabiting the Weixi area of the Baimaxueshan National Nature Reserve and the Mangkang area of the Mangkang Yunnan Snub-nosed Monkey National Nature Reserve. From 2017 to 2021, a total of 120 infrared cameras were deployed, yielding 21,813 image and video files containing *R. bieti*. Based on the PAE (Posture–Act–Environment) coding system, an ethogram and corresponding coding framework were constructed. In total, 16 postures, 84 acts, and 22 environmental factors were identified; 135 behaviors were defined and classified into 18 functional categories. On this basis, behavioral composition differences were quantitatively compared across seasons and geographic regions. The results showed that season significantly affected behavioral composition (F = 5.029, R^2^ = 0.255, *p* < 0.01). Four behavioral categories exhibited significant seasonal fluctuations: Locomotive behavior (χ^2^ = 12.97, *p* < 0.01), Ingestive behavior (χ^2^ = 12.04, *p* < 0.01), Resting behavior (χ^2^ = 26.18, *p* < 0.001), and Miscellaneous behavior (χ^2^ = 29.92, *p* < 0.001). Locomotive behavior peaked in summer, whereas Ingestive behavior was at its lowest and Resting behavior at its highest in autumn. Geographic region also significantly influenced behavioral composition (F = 2.808, R^2^ = 0.149, *p* < 0.01). These differences were mainly reflected in significantly lower Locomotive behavior (U = 13, *p* < 0.01) and higher Ingestive behavior (U = 13, *p* < 0.05) in the higher-elevation Mangkang group compared with the lower-elevation Weixi group. By integrating infrared camera with the PAE coding system, this study not only constructs an ethogram for *R. bieti* but also compares behavioral differences across seasonal and geographic dimensions. The findings demonstrate the comprehensive applicability of this technical pathway for macroscopic behavior recognition, frequency statistics, and difference analysis in wild primates, and provide fundamental data for further understanding the ecological driving mechanisms of behavioral plasticity and for differentiated conservation management of *R. bieti*.

## Simple Summary

The Yunnan snub-nosed monkey is one of the rarest primates in the world, living in the high mountains of southwestern China. Understanding how these monkeys behave in different seasons and locations is important for protecting them, but studying them is difficult because they live in remote areas and are easily disturbed by human presence. We used motion-triggered infrared cameras to monitor their natural activities over five years across two nature reserves. From more than 21,000 photos and videos, we built a detailed catalog of 135 distinct behaviors, such as feeding, moving, resting, and social interactions. We found that monkeys move most in summer, feed least and rest most in autumn, and adjust their activities to survive cold winters. We also discovered that monkeys living at higher elevations allocate more behavioral budget to feeding and less to moving than those at lower elevations, likely because colder conditions at higher elevations require greater energy intake and reduced energy expenditure to maintain thermal balance. This study shows that infrared cameras can effectively reveal how wildlife behavior changes across seasons and regions. Our findings provide practical information for designing targeted conservation measures, such as protecting key food plants in high-elevation areas, ensuring that different monkey populations receive the tailored protection they need.

## 1. Introduction

Behavioral plasticity is a core survival strategy enabling animals to cope with environmental heterogeneity [1,2,3]. When confronted with multiple challenges such as spatiotemporal fluctuations in food resources, social competition pressures, and habitat fragmentation, animals can buffer the directional selection pressures imposed by environmental change through adjustments in behavioral patterns [2,4]. Different geographic populations of the same species, exposed over long periods to divergent ecological conditions (e.g., climate, vegetation composition, predation pressure, and human disturbance), often exhibit systematic behavioral differentiation [5]. Such intraspecific behavioral variation represents both a direct manifestation of local adaptation and a crucial entry point for understanding a species’ ecological niche breadth and evolutionary potential [6,7]. As a fundamental task in animal behavior research, the construction and standardized coding of ethograms provide an ideal foundation and prerequisite for further investigation into the mechanisms, functions, and variations in animal behavior [8]. Since the establishment of ethology as a discipline in the 1930s, researchers have endeavored to construct systematic ethograms [9]. However, how to systematically classify and code animal behavior, which is continuous, fluid, and diverse in form, according to its ecological functions has remained a long-standing methodological challenge [10,11,12]. To address this dilemma, Jiang [11] proposed the PAE (Posture–Act–Environment) coding system, which decomposes behavior into three dimensions—posture, act, and environment—thereby preserving the full context in which behavior occurs while providing a structured interface for comparisons among different behaviors. This approach has been effectively applied to species such as the Milu (*Elaphurus davidianus*) and the Sichuan snub-nosed monkey (*Rhinopithecus roxellana*) [11,13].

As one of the mammalian groups exhibiting the highest behavioral complexity, primates have held particular theoretical and paradigmatic significance in behavioral ecology research [4,7,14]. Traditional behavioral observation has primarily relied on direct tracking and recording by researchers of animals. Although such methods possess irreplaceable advantages in individual identification and the details of social interactions [15], they are subject to three main limitations. First, the physical presence of the observer constitutes an environmental disturbance that can alter the natural behavioral expression of animals; in species with particularly high vigilance toward humans, this may lead to systematic biases in the recording of concealed or conflict behaviors [16]. Second, traditional behavioral observation methods require observers to maintain sustained high concentration for simultaneous recording over extended periods. Given the highly diverse social interactions and individual strategies of primates, intensive fieldwork leads to physical and visual fatigue, resulting in behavioral judgment errors that are difficult to correct [10]. Third, direct observation methods are constrained by the number of groups that can be followed and by geographic coverage, making it difficult to conduct systematic, simultaneous comparisons across multiple geographic populations [7,14]. Infrared camera technology enables continuous, round-the-clock monitoring without any disturbance to animals and can be deployed simultaneously at multiple sites, substantially expanding the geographic and temporal coverage of research [17,18]. Moreover, the images and videos can be replayed and verified repeatedly, avoiding the irreversible subjective judgment biases inherent in field observation and providing a technical safeguard for the consistency and reproducibility of behavioral coding [19]. In recent years, infrared cameras have been widely applied in wildlife population monitoring, activity rhythm analysis, and habitat use studies [18,20]. However, their use in the systematic construction of detailed ethograms for wild primates, and especially in the quantitative comparison of behavioral differences among different geographic populations, remains relatively rare.

The Yunnan snub-nosed monkey (*Rhinopithecus bieti*) is one of the non-human primates dwelling at the highest altitudes, with an elevational range reaching up to 4500 m [21,22,23]. Its distribution is restricted to a narrow belt of less than 20,000 km^2^ between the Jinsha and Lancang rivers in the Hengduan Mountains of China [24,25]. Previous studies have shown that *R. bieti* possesses a rich behavioral repertoire and a complex social structure, with its social organization exhibiting multi-level characteristics (breeding units–all-male units–band) [26,27,28]. Inter-individual interaction patterns are diverse, encompassing various social behaviors such as grooming, embracing, play, aggression, and reconciliation [24,26,27]. More importantly, the habitat of this species encompasses a complete vertical vegetation spectrum ranging from subtropical evergreen broad-leaved forest to cold-temperate dark coniferous forest, with an elevational span exceeding 2000 m. The climatic conditions, vegetation composition, and seasonal rhythms of food resources experienced by different geographic populations differ significantly [29,30]. For instance, populations in warm and humid low-elevation areas (ca. 2300 m) experience relatively balanced food resources throughout the year and a rich diversity of available vegetation types [29], whereas those in cold and arid high-elevation areas (>3500 m) face harsher temperature stress and pronounced seasonal food shortages [31,32]. Such steep ecological gradients are likely to drive adaptive differentiation among populations in behavioral dimensions such as foraging strategies, activity rhythms, and energy allocation [33]. These factors suggest that the behavioral patterns of *R. bieti* may exhibit significant inter-population geographic variation, which urgently needs to be empirically tested through a standardized behavioral coding system and systematic quantitative comparisons.

This study focused on *R. bieti* and conducted cumulative five-year infrared camera monitoring in the Weixi area of the Baimaxueshan National Nature Reserve and the Mangkang area of Mangkang Yunnan Snub-nosed Monkey National Nature Reserve. The objectives were to: construct a PAE coding system and a functional ethogram for wild *R. bieti* based on infrared camera images and videos; determine the relative frequencies and functional composition of various behavioral types; and then quantitatively test the effects of seasonal and geographic factors on behavioral composition, thereby identifying key behavioral types that exhibit significant differences. Through the above work, this study sought to further explore the applicability of infrared camera technology for constructing ethograms and quantitatively comparing behavioral differences in wild primates, to provide data support for understanding local behavioral adaptation in *R. bieti*, and to offer a scientific basis for the formulation of differentiated conservation management strategies for this species.

## 2. Materials and Methods

### 2.1. Research Area

**Figure 1:**
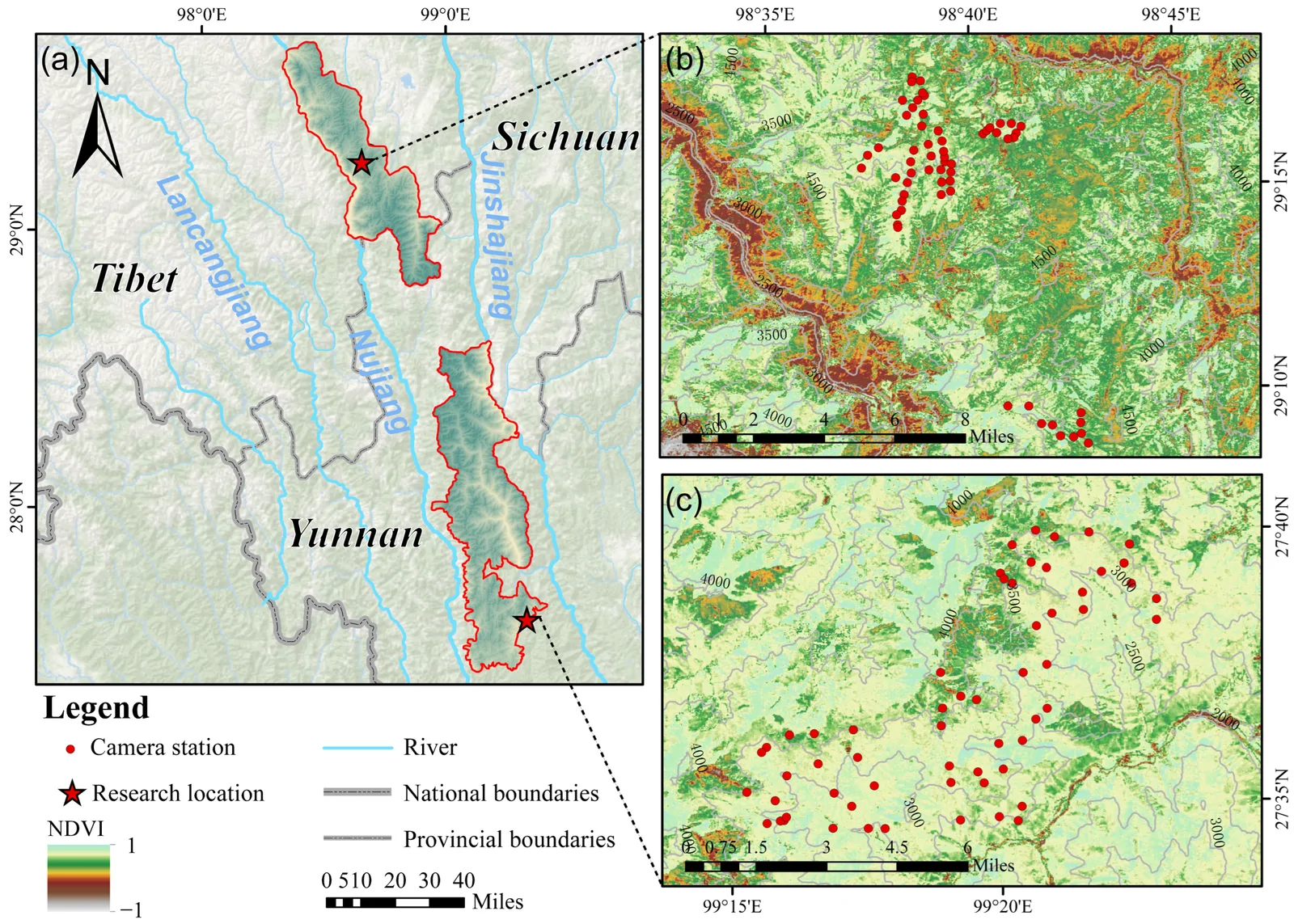
Study area and infrared camera survey design. (**a**) Geographic location of the study sites; (**b**) Infrared camera deployment sites in Mangkang area of Mangkang Yunnan Snub-nosed Monkey National Nature Reserve; (**c**) Infrared camera deployment sites in Weixi area of Baimaxueshan National Nature Reserve.

This study was conducted in the Mangkang area of the Mangkang Yunnan Snub nosed Monkey National Nature Reserve and the Weixi area within the Baimaxueshan National Nature Reserve (Figure 1). The two sites span a considerable elevational range and are influenced by different monsoon systems, resulting in marked climatic and vegetative differences. Weixi County (26°53′–28°02′ N, 98°54′–99°34′ E) has an average elevation of approximately 2340 m, a subtropical monsoon climate, a mean annual temperature of 11.4 °C, a mean annual precipitation of 915.5 mm, and a forest cover rate of 76.2%; the vegetation is dominated by subtropical and temperate mixed coniferous and broad-leaved forest. Mangkang County (28°37′–30°20′ N, 98°00′–99°05′ E) is situated on the southeastern margin of the Qinghai–Tibet Plateau, with an average elevation of about 3800 m and typical montane climatic characteristics; the mean annual temperature is approximately 3.5 °C, the mean annual precipitation is about 485 mm, and the forest cover rate is 70–80%. Pristine spruce and fir forests occur on shady slopes and in branch gullies, whereas sunny slopes are dominated by alpine shrubs.

### 2.2. Infrared Camera Survey Design and Data Processing

To obtain systematic records of natural behavior, we deployed infrared cameras (Ltl Acorn 6210MC, Zhuhai Ltl Acorn Electronics Co., Ltd., Zhuhai, China) across the primary activity ranges of *R. bieti* in both the Weixi and Mangkang areas. The Weixi area encompassed three monitoring sites—Gehuaqing, Xiangguqing, and Yalongqing—where 60 cameras were deployed, with monitoring conducted from January 2017 to March 2021. In the Mangkang area, 60 cameras were deployed, and monitoring was conducted from January 2020 to December 2021. A total of 120 cameras were deployed across the two areas (Table 1).

Prior to deployment, transects were delineated within areas of high-frequency use by monkey groups based on historical patrol records of the reserves, and one camera was installed approximately every 500 m along the transects to ensure independence of detection among cameras [34]. During deployment, locations with dense traces of *R. bieti*, trail intersections, and water sources were prioritized for camera installation. Each camera was mounted at a height of 50–80 cm above the ground, and the angle was adjusted appropriately to capture data at a certain height [35]. The cameras were positioned to ensure a wide field of view and to avoid direct sunlight. After installation, information including camera ID, date and time, latitude and longitude, elevation, slope, aspect, and vegetation type was recorded. Camera settings were configured as follows: upon each trigger, three photos were taken and a 15 s video was recorded; the trigger interval was set to 1 s; the camera clock was set to 24 h format; and sensitivity was set to “medium” [36]. Every 3–4 months, the working status of the cameras was checked, data were retrieved, and batteries and memory cards were replaced. Each time the raw infrared camera data were retrieved, they were numbered according to the camera location and stored separately by area.

The behavioral identification work was conducted from October 2024 to April 2026. Prior to data processing, a complete backup of the raw data was first created, and all subsequent operations were performed on the backup copies to ensure the integrity and traceability of the original data. Subsequently, blank, falsely triggered, and invalid images and videos were removed. Species identification was performed on the valid photos, and all images and videos containing *R. bieti* were screened out for subsequent behavioral identification and coding analysis.

### 2.3. Definition and Coding of Behavior

Following the PAE coding system proposed by Jiang (2000) [11], the behavior of *R. bieti* was decomposed into three basic units: Posture, Act, and Environment. “Posture” refers to the position and configuration of the main body structures of an individual over a certain period of time; “Act” refers to the localized displacement, relaxation, or bending of body parts induced by muscle group movements over a short duration; “Environment” refers to the abiotic environment (e.g., vegetation type, topography, weather) and biotic environment (e.g., group composition, inter-individual relationships) in which the behavior occurs. Behavioral coding followed the set relationship *b_i_* = *p_i_* ∩ *a_i_* ∩ *e_i_*, where *b_i_*, *p_i_*, *a_i_*, and *e_i_* represent the codes for behavior, posture, act, and environment, respectively [37].

During the behavioral identification process, the images and videos were first viewed and compared repeatedly. Behavioral types were preliminarily classified according to behavioral function. Subsequently, the posture, act, and environmental elements involved in each behavior were decomposed one by one. Acts that were morphologically similar but differed substantially in ecological function were coded separately. Existing studies [13,38] were consulted for nomenclature and definitions, and behaviors were classified according to their ecological functions. For multiple individuals appearing simultaneously in a single photo, the posture and act information of each individual was identified and recorded separately, and the behavior of each individual was coded as an independent entry. In the continuous videos recorded by the infrared cameras, the same individual could exhibit continuous and variable sequences of acts. Each 15 s video was analyzed through second-by-second playback and segmented into discrete, codable behavioral units to ensure the completeness and accuracy of behavioral coding and to reduce subjective judgment bias in behavioral state assessment.

In this study, the minimum duration of a behavioral event was defined as 1 s. When a posture–act combination was stable and functionally unitary and persisted for more than 1 s, it was recorded as an independent behavioral event. When the posture or act changed, and the change met the criteria for defining a behavioral event, it was regarded as a behavioral event switch and coded separately. Transitional postures (e.g., the semi-squatting state during the transition between standing and sitting) were not coded until the individual adopted the next standard posture.

### 2.4. Statistical Analysis of Behavioral Differences

Taking into account the camera trigger settings, we cleaned and standardized the raw behavioral records by removing duplicate images and videos records generated by consecutive triggers within the same behavioral sequence. Behavioral records in which the three elements—posture, act, and environment—could all be unambiguously identified and the focal individual was clearly discernible were retained as valid samples. Each valid behavioral record was then converted into the corresponding behavioral category according to the coding system established in Section 2.3 “Definition and Coding of Behavior” To eliminate the imbalance in observation opportunities caused by differences in effective monitoring duration across monitoring sites, each calendar month was treated as an independent monitoring unit. The percentage of the frequency of a given behavior type relative to the total behavioral frequency in that month was calculated as the relative frequency of that behavior for the month [15,39], thereby enabling exploration of behavioral differences in *R. bieti* across seasons and geographic regions.

For the analysis of seasonal behavioral differences, the Northern Hemisphere meteorological season classification (spring: March–May; summer: June–August; autumn: September–November; winter: December–February) was adopted [40]. The monthly behavioral frequencies from each area were grouped by season. Taking the behavioral frequency vector of each monitoring unit as a sample, a Bray–Curtis dissimilarity matrix was calculated among samples to quantify the overall difference in behavioral composition. PERMANOVA was used to test the effect of seasonal variation on behavioral composition. The null hypothesis of the global test was that there were no differences in behavioral composition among seasons; pseudo-F statistics and *p*-values were obtained through 999 random permutations [41]. When the global test was significant, pairwise comparisons of behavioral composition between seasons were conducted by repeating the PERMANOVA procedure. To identify which specific behaviors differed significantly among seasons, the Kruskal–Wallis test was applied to each behavior independently, and we use the Mann–Whitney U test to see how behavior changes across seasons.

For the analysis of geographic variation in behavior, we used the Mann–Whitney U test to compare the overall behavior composition of Yunnan snub-nosed monkeys between the Weixi and Mangkang regions, and then used the same method to check the differences for each type of behavior between the two locations.

The above analytical methods accommodate non-normal and heteroscedastic data [42,43]. All statistical analyses were performed in R 4.2.3, with the significance level uniformly set at *p* = 0.05.

## 3. Results

### 3.1. PAE Coding System and Ethogram of R. bieti

#### 3.1.1. Posture Codes

Through systematic collation and repeated observation of the infrared camera images and videos, combined with definitions of primate postures from existing studies, this study identified a total of 16 basic postures of *R. bieti*, which could be classified into three categories: stationary postures, locomotor postures, and care postures (Table 2). Compared with the posture classification for *R. bieti* by Li [38], this study added and recorded two new postures, “Climbing” and “Sprawling”. “Climbing” is used to describe the vertical movement of an individual on a tree trunk; “Sprawling” is a stationary prone posture in which the individual’s abdomen is pressed close to the substrate with all four limbs outstretched and touching the ground, and is mainly observed in the solicitation behavior of adult females and the submissive behavior of juveniles.

#### 3.1.2. Act Codes

This study categorized movements according to their anatomical origins into six distinct categories: head and neck, mouth, eyes, ears and nose, face, limbs, and hindquarter, resulting in the identification of a total of 84 unique movements (Table 3). Limbs movements represented the largest proportion, comprising 33 types (39.29%), which included various actions such as grasping, pulling, running, and jumping, as well as manipulative activities. Mouth movements followed, accounting for 19 types (22.62%), primarily associated with feeding and vocal communication. Movements in the hindquarter comprised 16 types (19.05%), while head and neck movements included 8 types (9.52%). Finally, movements of the eyes, ears, and nose accounted for 6 types (7.14%), and facial movements represented 2 types (2.38%).

#### 3.1.3. Environment Codes

Environmental Information was collected based on the field records completed during infrared camera deployment, integrated with the specific habitat characteristics of the behavior occurrence observed in the images and videos. The environment in which *R. bieti* behaviors occurred was divided into two major categories: biotic environment and abiotic environment, yielding a total of 22 codes (Table 4).

#### 3.1.4. PAE Coding System and Ethogram

**Figure 2:**
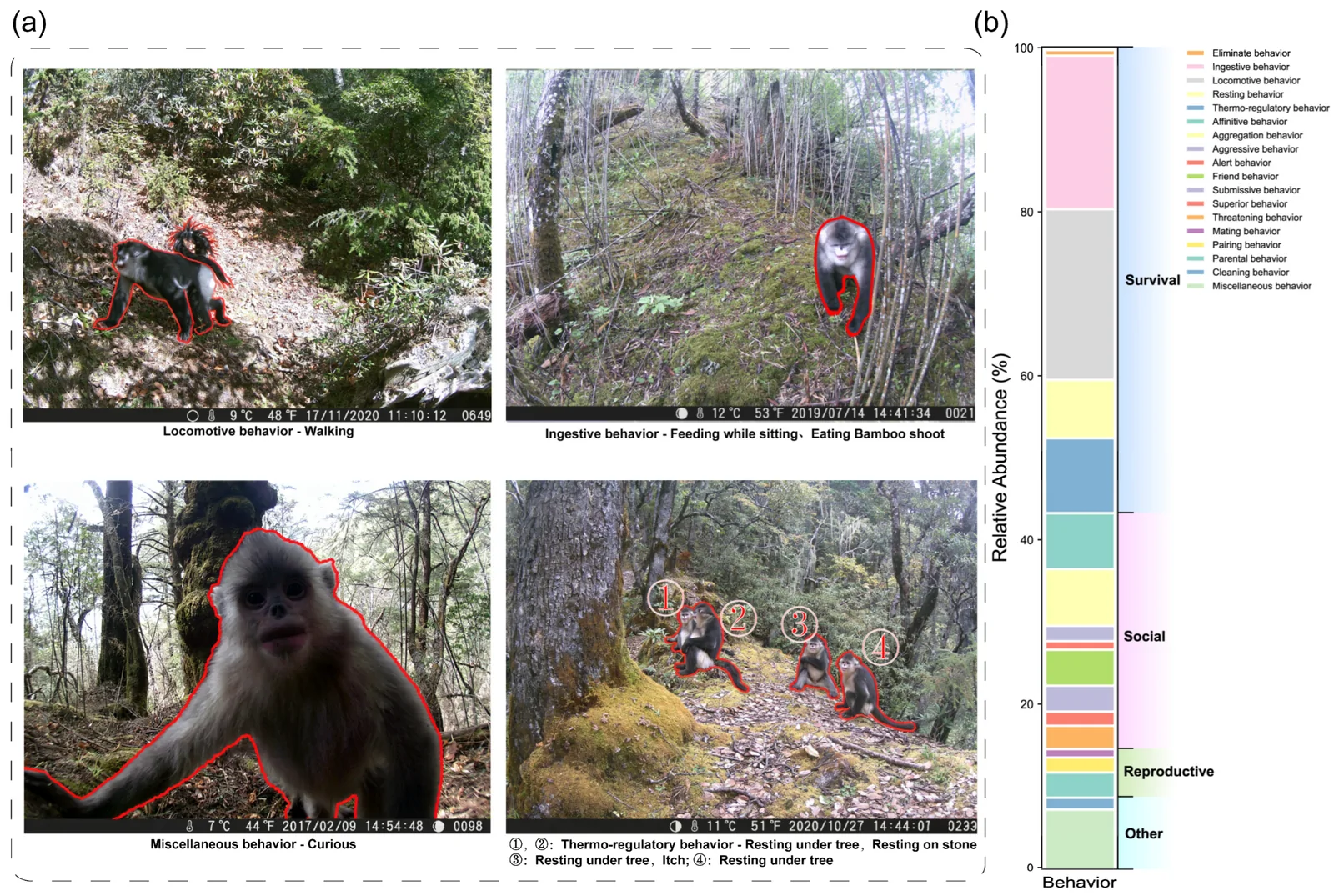
Schematic illustration of selected behaviors and frequency proportions of functional behavioral categories in *R. bieti*. (**a**) Schematic illustration of selected behaviors; (**b**) stacked bar chart showing the proportion of each behavioral category relative to the total behavioral frequency.

Through systematic observation and coding of the images and videos, a total of 135 behaviors were recorded for *R. bieti*. Based on ecological function, they were classified into four major categories—survival behaviors (Locomotive, Ingestive, Eliminate, Thermo-regulatory, Resting), social behaviors (Superior, Threatening, Alert, Aggressive, Submissive, Friendly, Affinitive, Aggregation), reproductive behaviors (Pairing, Mating, Parental), and other behaviors (Cleaning, Miscellaneous)—comprising 18 subcategories (Table S1). In terms of overall behavioral composition, survival behaviors occurred most frequently (56.50%). Locomotion and Ingestive were the predominant activity types, together accounting for over 40% of all behaviors. Social behaviors were the second most frequent, constituting 28.74%, with Aggregation (6.86%), Affinitive (6.84%), and Friendly (4.40%) occurring at relatively high frequencies. Reproductive behaviors accounted for 5.93% in total, and other behaviors accounted for 8.83% (Figure 2).

### 3.2. Behavioral Difference Comparison

#### 3.2.1. Seasonal Behavioral Difference Comparison

**Figure 3:**
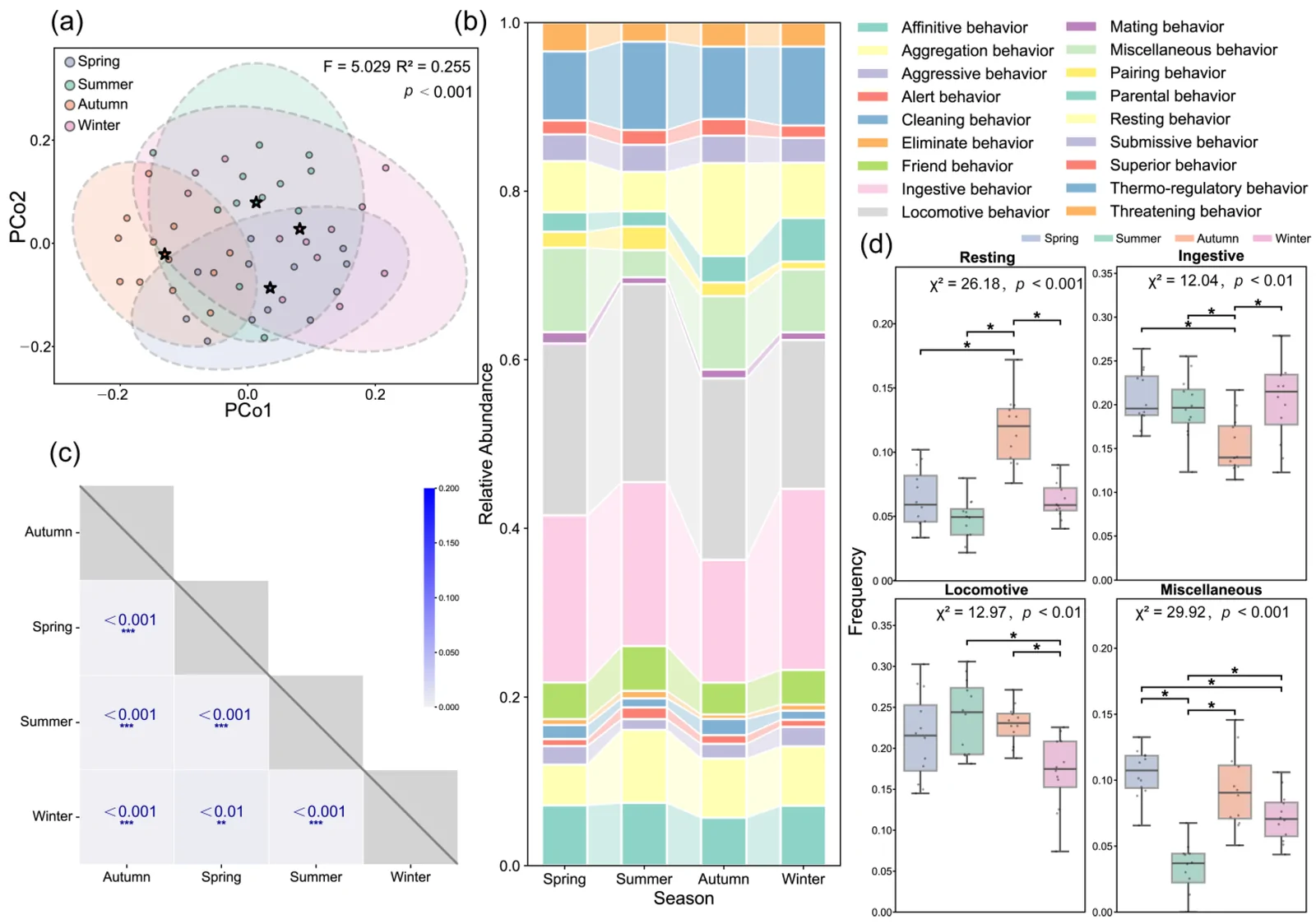
Seasonal variation in behavioral composition of *R. bieti*. (**a**) PCoA plot showing seasonal separation; (**b**) Relative frequency of each behavioral category across seasons; (**c**) Heatmap of pairwise PERMANOVA comparisons; (**d**) Boxplots of behaviors with significant seasonal differences. Significance levels are indicated as follows: *** *p* < 0.001; ** *p* < 0.01; * *p* < 0.05.

The PERMANOVA results showed that seasonal variation had a significant effect on the behavioral composition of *R. bieti* (F = 5.029, R^2^ = 0.255, *p* < 0.01; Figure 3a). Further pairwise comparisons among the four seasons revealed that behavioral composition differed significantly between all six season pairs (*p* < 0.01; Figure 3c), with the greatest difference observed between summer and autumn (F = 7.3533, R^2^ = 0.2505, *p* < 0.001) and the smallest between spring and winter (F = 2.6131, R^2^ = 0.1062, *p* < 0.01).

Kruskal–Wallis rank sum tests on individual behavioral types revealed statistically significant seasonal differences in the frequency distributions of four behaviors (Figure 3d). In descending order of chi-square (χ^2^) values: Miscellaneous behavior (χ^2^ = 29.92, *p* < 0.001), Resting behavior (χ^2^ = 26.18, *p* < 0.001), Locomotive behavior (χ^2^ = 12.97, *p* < 0.01), and Ingestive behavior (χ^2^ = 12.04, *p* < 0.01). The differences in Resting behavior and Miscellaneous behavior reached highly significant levels, indicating that these two types of behavior were most strongly affected by seasonal variation. In terms of seasonal patterns, Locomotive behavior was significantly higher in summer than in other seasons; and autumn was characterized by Ingestive behavior being the lowest of the year but Resting behavior the highest. In contrast, the remaining behavioral types, such as Affinitive behavior and Friend behavior, showed no significant seasonal differences.

#### 3.2.2. Geographic Behavioral Difference Comparison

**Figure 4:**
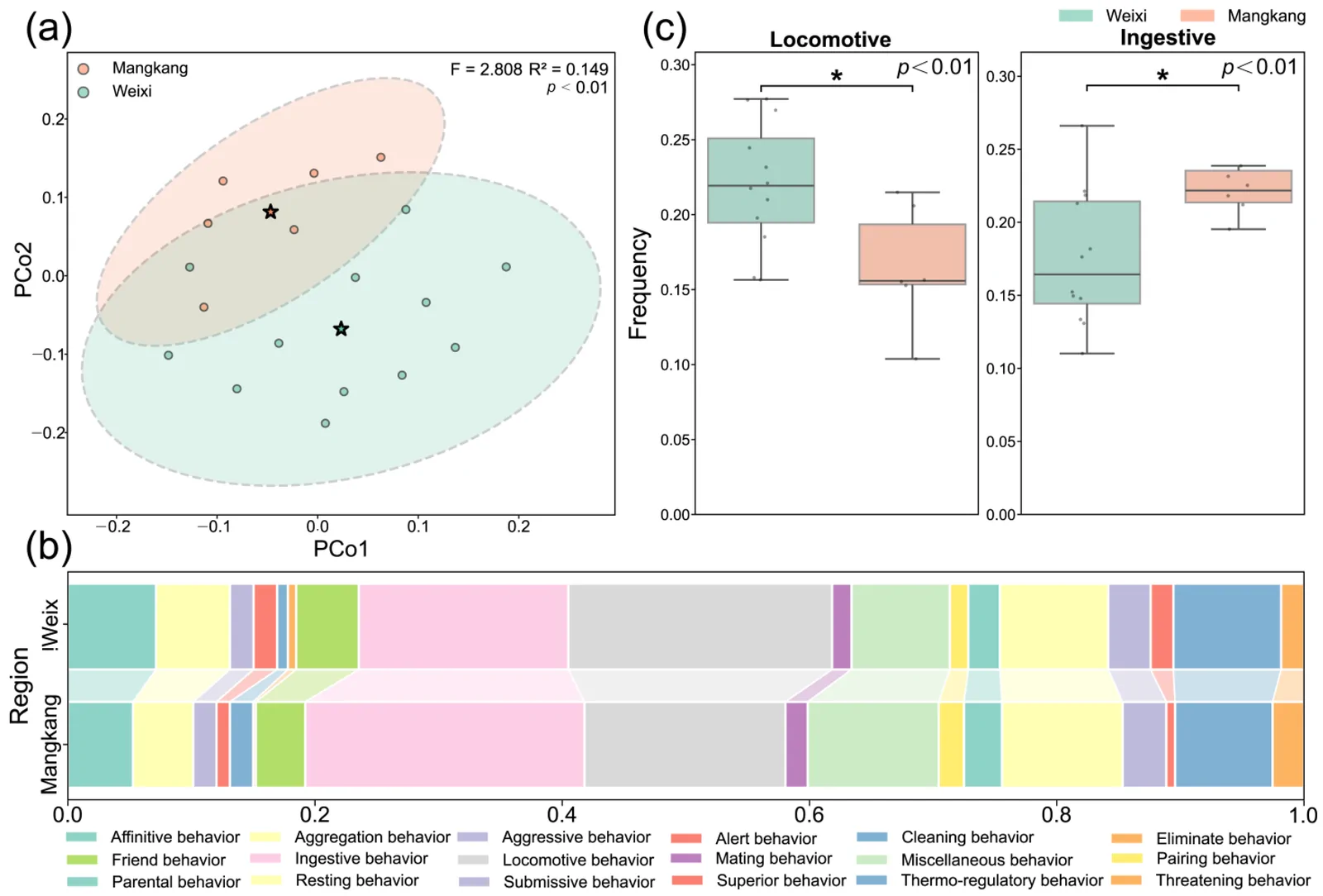
Geographic variation in behavioral composition of *R. bieti*. between Weixi and Mangkang. (**a**) PCoA plot showing regional separation; (**b**) Relative frequency of each behavioral category in the two regions; (**c**) Boxplots of behaviors with significant inter-regional differences. Significance levels are indicated as follows: * *p* < 0.05.

Geographic region also significantly influenced the behavioral composition of *R. bieti* (PERMANOVA, F = 2.8082, R^2^ = 0.1493, *p* < 0.01; Figure 4a). When each behavioral type was compared individually between the two areas using the Mann–Whitney U test, only Locomotive behavior (U = 13, Z = 0.75, *p* < 0.01) and Ingestive behavior (U = 13, Z = −0.64, *p* < 0.05) showed significant differences (Figure 4c). Specifically, the Weixi group exhibited a significantly higher frequency of Locomotive behavior but a lower frequency of Ingestive behavior than the Mangkang group. No other behavioral types differed significantly between the two areas.

## 4. Discussion

### 4.1. Applicability and Biases of Infrared Camera in PAE Coding System Construction

For a rare and endangered primate such as *R. bieti* that inhabits high-altitude mountainous regions and is extremely sensitive to human activity, infrared camera technology offers unique observational advantages for constructing a PAE coding system. This technique enables continuous, round-the-clock monitoring without observer disturbance and can be deployed simultaneously at multiple sites, greatly expanding the spatial and temporal coverage of behavioral sampling [18,19]. Moreover, infrared camera images and videos can be stored long-term and reviewed repeatedly, thereby reducing behavioral judgment errors that may arise from rapid decision-making during field observation, and providing a technical safeguard for the standardization and reproducibility of behavioral coding [19].

However, this technique also has inherent limitations. First, the field of view of a single camera is limited, and its capacity to resolve fine-scale social interactions and inter-individual spatial relationships is weaker than that of direct observation [44]. Second, camera triggering depends on animal movement, leading to a systematic underestimation of sustained stationary behaviors [18]. This is clearly evidenced by the fact that the relative frequency of Resting behavior in the present study (7.12%) was markedly lower than the 20.9% reported by Li, Ren et al. (2013) based on instantaneous scan sampling [45]. Third, owing to limitations in image resolution and camera angle, subtle communication behaviors such as vocalizations and facial expressions are difficult to identify accurately. Furthermore, event-frequency data from infrared cameras and time-budget data from direct observation belong to different measurement dimensions; no direct linear conversion relationship exists between them, and this distinction must be explicitly recognized during interpretation [18]. Therefore, the event-frequency data derived from infrared cameras are not intrinsically superior to time-budget data from traditional observation; rather, they excel in data retrospection, disturbance-free monitoring, and simultaneous multi-site comparisons. The two methodological approaches are complementary rather than mutually exclusive.

### 4.2. Seasonal Behavioral Differences in R. bieti

When facing seasonal environmental fluctuations, primates typically maintain their annual energy balance through structural adjustments of behavioral time budgets [46,47,48]. In the present study, PERMANOVA results showed that seasonal variation significantly affected the overall behavioral composition of *R. bieti* (F = 5.029, R^2^ = 0.255, *p* < 0.01). The seasonal alternation of habitat quality, particularly cyclical changes in food resource abundance and climatic conditions, represents the dominant factor driving behavioral variation [32]. In terms of specific seasonal patterns, summer marks the peak of the plant growing season, when the variety of edible items reaches its annual maximum. *R. bieti* responds by increasing Locomotive behavior to expand foraging ranges and exploit dispersed high-quality food resources, a pattern consistent with that reported by Grueter et al. (2013) [32]. Meanwhile, the mating frequency of *R. bieti* peaks in summer [49], and the increased reproductive investment, particularly mate-searching and courtship activities may also directly contribute to the elevated Locomotive behavior during this season. Autumn is the concentrated fruit maturation period, during which energy-dense foods enable individuals to meet their energy requirements within a shorter feeding duration [50]. Winter imposes the dual pressures of food scarcity and low-temperature stress. *R. bieti* adopts an energy management strategy combining “increasing energy intake” and “reducing energy expenditure”: on the one hand, it increases feeding investment to compensate for the energy demands imposed by declining food quality and elevated basal metabolism; on the other hand, it reduces non-essential activities to lower energy consumption [51,52]. This “high feeding, low locomotion” pattern has also been documented in the Sichuan snub-nosed monkey [53] and in the high-altitude Shortridge’s langur (*Trachypithecus shortridgei*) [54], suggesting that it may represent a conserved adaptive strategy of high-altitude primates for coping with extreme environments. The study by Li [45] based on instantaneous scan sampling also supports this pattern: feeding time was longest in winter (41.5%), whereas moving time was highest in summer (32.8%). The present study provides an independent methodological validation of this behavioral budget allocation pattern at the behavioral frequency level; although the two methods differ in measurement dimensions, the overall seasonal trends are highly consistent.

### 4.3. Geographic Differentiation of Behavioral Differences in R. bieti

Geographic variation in behavior is widespread among primates [27,29]. The core behavioral differences between the Mangkang and Weixi groups were manifested as significantly lower Locomotive behavior (U = 13, *p* < 0.01) and significantly higher ingestive behavior (U = 13, *p* < 0.05) in Mangkang. This pattern is jointly driven by the elevational gradient and geographic heterogeneity in food resources [27]. Elevation is likely the primary ecological factor. The Mangkang area lies at the northernmost limit of the distribution of *R. bieti*, with a markedly higher average elevation than Weixi; the monkeys there face lower temperatures, thinner oxygen, and more severe energetic stress [32], they need a lot of metabolic energy to maintain their core body temperature and might spend more time eating just to meet the extra metabolic demands of staying warm, while also cutting down on unnecessary activities to save energy [32]. A systematic analysis of 50 colobine populations worldwide by Kraus and Strier (2022) showed that snub-nosed monkeys (Rhinopithecus) are the only colobine taxon in which feeding time increases significantly with rising elevation and decreasing temperature [55], reflecting a unique energy-maximizing adaptive strategy in high-altitude environments. The present study provides intraspecific empirical support for this cross-species pattern at the behavioral frequency level.

Food resource availability and spatial distribution constitute another important factor driving geographic behavioral differentiation [27,32]. The diet of *R. bieti* differs significantly among geographic populations [32]. Previous studies have shown that the diet of *R. bieti* in the Mangkang area is dominated by lichens throughout the year, which can account for 47–64% of the diet and can be exploited without frequent movement [21,56]. In contrast, the Weixi area is situated in subtropical mixed coniferous and broad-leaved forest, where food resources such as young leaves, fruits, and bamboo shoots exhibit a patchy distribution and entail higher travel costs [45]. This interpretation is consistent with the findings of Ren et al. (2010) on geographic populations of the Sichuan snub-nosed monkey—differences in dietary composition among populations are mainly attributed to differences in elevational zone overlap and forest types [57]. In addition, group size also affects behavior adjustments. Previous studies have shown that larger groups tend to move longer distances daily [58], and the significantly larger monkey groups in the Weixi area also mean a higher need for resources, which might lead to more movement behavior. It is noteworthy that elevation and food resources do not act independently; high elevation shortens the plant growing season, fundamentally limiting the diversity and abundance of food resources, which in turn elevates the investment in feeding behavior [33,55]. These findings provide new quantitative evidence for understanding geographic behavioral differentiation in snub-nosed monkeys.

### 4.4. Conservation Implications and Research Prospects

The findings of this study have direct relevance for the conservation management of *R. bieti*. First, the marked increase in feeding behavior in the high-elevation Mangkang population indicates that this population faces greater energy acquisition pressure; the protection and restoration of key food plants, such as lichens, in its habitat should be treated as a priority. Second, the behavioral differences among geographic populations suggest that a uniform management strategy may be inadequate to meet the actual needs of each area, and that differentiated conservation plans should be formulated according to the ecological characteristics and behavioral profiles of each community. Third, the seasonal restructuring of behavior implies that conservation management must incorporate a temporal dimension: during the food-scarce winter and the reproductive-energy-intensive summer, the energy demands and space-use patterns of the monkey groups undergo structural changes, and the timing of conservation interventions should be adjusted accordingly.

From a methodological perspective, the “Infrared camera + PAE coding system” framework established in this study can be extended to the non-invasive behavioral monitoring of other rare and endangered primates, providing a reusable technical pathway for standardized comparisons of inter- and intraspecific behavioral variation. Future research could further integrate GPS tracking, fecal metabolomics, and microclimate data to reveal, from an energetic perspective, the adaptive thresholds of behavioral plasticity and the mechanisms underlying responses to climate change.

## 5. Conclusions

This study establishes a comprehensive PAE ethogram for *R. bieti* and demonstrates that its behavioral patterns vary significantly across seasons and geographic regions. Seasonally, *R. bieti* increased locomotion in summer, reduced feeding with elevated resting in autumn, and a “high feeding, low movement” pattern in winter, which was driven by food availability, reproductive investment, and thermoregulatory demands. Geographically, the higher elevation Mangkang population exhibits greater feeding effort and reduced locomotion compared with the lower elevation Weixi population, reflecting adaptations to colder temperatures, lichen-dominated diets, and larger group sizes. This study provides quantitative evidence for understanding the ecological driving mechanisms of behavioral plasticity in *R. bieti* and lays a behavioral foundation for the development of differentiated conservation management strategies for this species.

## Figures and Tables

**Table 1:** Overview of the Deployment of Infrared Cameras and Data Collection.

Research Location	Weixi			Mangkang
	Gehuaqing	Xiangguqing	Yalongqing	
Deployment period	January 2017–March 2021	January 2017–March 2021	January 2017–March 2021	January 2020–December 2021
Number of cameras	20	20	20	60
Total infrared camera days	29,598	29,986	30,151	41,160
Total photographs	118,045	126,454	132,660	112,463
Images containing *R. bieti*	2602	5927	5564	3474
Videos containing *R. bieti*	706	1496	1231	813

**Table 2:** Posture codes for *R. bieti*.

Type	Postures	Code
Static Posture	Standing	1
	Rearing	2
	Sitting	3
	Groveling	4
	Lying	5
	Crouching	6
	Sprawling	7
Exercise Posture	Hanging	8
	Climbing	9
	Walking	10
	Running	11
	Jumping	12
	Mounting	13
Care Posture	Cradling	14
	Carrying	15
	Nursing	16

**Table 3:** Act codes for *R. bieti*.

Part	Action	Code	Part	Action	Code
Head and neck	Swing	1	Limbs	Scratch	43
	Shake head	2		Strip	44
	Turn head	3		Haul	45
	Extend forward	4		Drag	46
	Turn left	5		Pinch	47
	Turn right	6		Push	48
	Raise head	7		Shake	49
	Lower head	8		Embrace	50
Mouth	Gnaw	9		Climb	51
	Chew	10		Standing	52
	Kiss	11		Hindlegs standing	53
	Lick	12		Step forward	54
	Bite	13		Step backward	55
	Hold in mouth	14		Step diagonally	56
	Open mouth	15		Running	57
	Showing teeth	16		Jumping	58
	Tongue out	17		Forelegs hit	59
	Swallow	18		A foreleg bends	60
	Close mouth	19		Forelegs bent	61
	Suck	20		A foreleg straightens	62
	Yawn	21		Forelegs straight	63
	Bleat	22		A hindleg bends	64
	Scream	23		Hindlegs bent	65
	Roar	24		A hindleg straightens	66
	Howl	25		Hindlegs straight	67
	Close eyes	26		Shiver	68
	Blink	27	Hindquarter	Plane	69
Eyes, ears and nose	Stare	28		Stretch	70
	Watch	29		Bend	71
	Exhale	30		Turn left	72
	Inhale	31		Turn right	73
	Sniff	32		Tail raised	74
	Touch with muzzle	33		Tail shake	75
Face	Frown	34		Tail down	76
	Relaxation	35		Defecate	77
Limbs	Slapping	36		Mount	78
	Rub	37		Creep	79
	Press	38		Erect	80
	Hold	39		Insert	81
	Pull into arms	40		Vellicate	82
	Grasp	41		Ejaculate	83
	Groom	42		Withdraw	84

**Table 4:** Environment codes for *R. bieti*.

Environment	Biotic (E1)	Abiotic (E2)	Code
Arbor	√		1
Shrub	√		2
Grass	√		3
Bamboo Grove	√		4
Stone		√	5
Bare ground		√	6
Snowfield		√	7
Water		√	8
Ridge		√	9
Hillside		√	10
Valley		√	11
Solar slope		√	12
Lunar slope		√	13
Semi-solar slope		√	14
Male	√		15
Female	√		16
Infant	√		17
Juvenile	√		18
Family group	√		19
Mother–child group	√		20
Mixed group	√		21
Single	√		22

√ indicates that it belongs to this type.

## Data Availability

The original contributions presented in this study are included in this article. Further inquiries can be directed to the corresponding author.

## Supplementary Materials

The following supporting information can be downloaded at: https://www.mdpi.com/article/10.3390/biology15171509/s1, Table S1: The ethogram of *R. bieti* based on the PAE coding system.
