# Supplementary material for: PAE Coding System and Ethogram of Wild Yunnan Snub-Nosed Monkeys with Behavioral Comparisons Based on Infrared Camera Data
Source: Biology (Basel). 2026 Sep 3;15(17):1509. doi: 10.3390/biology15171509 (PMC13564757; doi:10.3390/biology15171509)
Supplement: Supplementary file 1 [file biology-15-01509-s001.zip › biology-4463444-supplementary.pdf]

Supplementary File

Table S1. The ethogram of *R. bieti* based on the PAE coding system.

Behaviors	Adult male	Adult female	Juvenile	Infant	Season	Order code	Posture	Act	Environment
**1. Ingestive behavior**									
Foraging	+++	+++	+++	+++	Y	1	10	4, 5, 6, 7, 8, 45, 53, 55	1, 2, 3, 4, 6
Begging food				+	Y	2	1, 3	4, 7, 15	15, 16, 18, 21, 22
Feeding while standing	++	++	+++	+	Y	3	1, 2, 3, 4, 8, 10	7, 8, 9, 10, 13, 14, 15, 16, 17, 18, 19, 33, 42, 45, 53, 60, 61, 63	1, 2, 4
Feeding while walking	++	++	+		Y	4	10	5, 6, 7, 8, 9, 10, 13, 14, 15, 16, 17, 18, 19, 33, 42, 45, 56, 61, 63	2, 3, 4, 6
Feeding while kneeling down	+	++	+++		Y	5	4	5, 6, 9, 10, 12, 13, 14, 15, 16, 17, 18, 19, 33, 42, 45, 61, 62, 63, 64, 66, 68	2, 3, 4
Feeding while rearing forelegs	++		+		Y	6	2	7, 8, 9, 10, 13, 14, 15, 16, 17, 18, 19, 40, 42, 46, 54, 61, 62, 63, 64, 66, 68, 71	1, 2, 3, 6
Feeding while sitting	+++	+++	+++	++	Y	7	3	7, 8, 9, 10, 13, 14, 15, 16, 17, 18, 19, 42, 47, 61, 62, 63, 64, 66	1, 2, 3
Feeding while hanging	+	+	++		Y	8	8	7, 8, 9, 10, 13, 14, 15, 16, 17, 18, 19, 20, 40, 42, 46, 47, 61, 63, 64, 68	1, 2
Feeding while jumping	+		+		Y	9	12	7, 8, 9, 10, 13, 14, 15, 16, 17, 18, 19, 20, 42, 46, 47, 50, 54, 59, 61, 62, 63, 64, 66, 68	1, 2
Eating root	+++	+++	+++	++	Y	10	1, 2, 3, 4, 5, 8, 10, 11, 12	7, 8, 9, 10, 13, 15, 16, 17, 18, 19, 33, 34, 42, 45, 47, 53, 54, 55, 56, 58, 59, 61, 62, 63, 64, 66, 68	1, 2, 3
Eating young stem	++	++	+	++	W, Sp	11	1, 2, 3, 4, 5, 8, 10, 11, 12	7, 8, 9, 10, 13, 15, 16, 17, 18, 19, 33, 34, 42, 45, 47, 53, 54, 55, 56, 58, 59, 61, 62, 63, 64, 66, 68	1, 2, 3
Eating leaf	+++	+++	+++	++	Y	12	1, 2, 3, 4, 5, 8, 10, 11, 12	7, 8, 9, 10, 13, 15, 16, 17, 18, 19, 33, 34, 42, 45, 47, 53, 54, 55, 56, 58, 59, 61, 62, 63, 64, 66, 68	1, 2, 6
Eating flower	+++	+++	+++	+	Sp, S	13	1, 2, 3, 4, 5, 8, 10, 11, 12	7, 8, 9, 10, 13, 15, 16, 17, 18, 19, 33, 34, 42, 45, 47, 53, 54, 55, 56, 58, 59, 61, 62, 63, 64, 66, 68	1, 2

Behaviors	Adult male	Adult female	Juvenile	Infant	Season	Order code	Posture	Act	Environment
Gnawing bark	+ + +	+ +	+ +		W	14	1, 2, 3, 4, 5, 8, 10, 11, 12	7, 8, 9, 10, 13, 15, 16, 17, 18, 19, 33, 34, 42, 45, 47, 53, 54, 55, 56, 58, 59, 61, 62, 63, 64, 66, 68	1, 2
Eating fruit or seed	+ +	+ + +		+	S, A	15	1, 2, 3, 4, 5, 8, 10, 11, 12	7, 8, 9, 10, 13, 15, 16, 17, 18, 19, 33, 34, 42, 45, 47, 53, 54, 55, 56, 58, 59, 61, 62, 63, 64, 66, 68	1, 2
Eating bud	+ + +	+ + +	+ +	+ + +	W, Sp	16	1, 2, 3, 4, 5, 8, 10, 11, 12	7, 8, 9, 10, 13, 15, 16, 17, 18, 19, 33, 34, 42, 45, 47, 53, 54, 55, 56, 58, 59, 61, 62, 63, 64, 66, 68	1, 2, 4
Eating fungus	+ +	+ +	+ +		S	17	1, 2, 3, 4, 5, 8, 10, 11, 12	7, 8, 9, 10, 13, 15, 16, 17, 18, 19, 33, 34, 42, 45, 47, 53, 54, 55, 56, 58, 59, 61, 62, 63, 64, 66, 68	1, 2, 3, 4
Eating insect	+ +	+	+		Y	18	1, 2, 3, 4, 5, 8, 10, 11, 12	7, 8, 9, 10, 13, 15, 16, 17, 18, 19, 33, 34, 42, 45, 47, 48, 53, 54, 55, 56, 58, 59, 61, 62, 63, 64, 66, 68	1, 2, 3, 4, 6
Eating Bamboo shoot	+ +		+ +		Sp, S	19	1, 2, 3, 4, 5, 8, 10, 11, 12	7, 8, 9, 10, 13, 15, 16, 17, 18, 19, 33, 34, 42, 45, 47, 53, 54, 55, 56, 58, 59, 61, 62, 63, 64, 66, 68	4
Sucking				+ +	Y	20	1, 2, 3, 4, 14	5, 6, 7, 12, 13, 14, 15, 17, 18, 20, 51	17, 19, 20
Chewing	+ + +	+ + +	+ + +	+ +	Y	21	1, 2, 3, 4, 5, 8, 10, 11, 12	5, 6, 7, 8, 10, 15, 18, 19	1, 2, 3, 4, 5, 6, 7
Drinking	+ + +	+ + +	+ + +	+ + +	Y	22	1, 4	8, 12, 15, 18, 20, 53, 54, 64, 68	8
Eating soil		+	+ +		Y	23	1, 2, 3, 4, 10, 11	8, 9, 10, 12, 15, 17, 18, 19, 42, 45, 48, 53, 54, 55, 61, 63, 66, 68	3, 6
Eating snow	+	+	+		W	24	1, 2, 3, 4, 10, 11	8, 9, 10, 12, 15, 17, 18, 19, 42, 45, 48, 53, 54, 55, 61, 63, 66, 68	7
**2.Eliminate behavior**									
Defecating while walking	+		+		Y	25	10, 11, 12	36, 53, 55, 58, 59, 64, 68, 75, 78	4, 5, 6, 9, 10, 11
**3.Thermo-regulatory behavior**									
Resting on branch	+ + +	+ + +	+ + +	+ +	Y	26	3, 4, 5	5, 6, 7, 8, 19, 21, 27, 36, 43, 51, 61, 62, 63, 64, 65, 66, 67, 68	1

Behaviors	Adult male	Adult female	Juvenile	Infant	Season	Order code	Posture	Act	Environment
Resting under tree	+ + +	+ + +	+ + +	+ + +	Y	27	3, 4, 5	5, 6, 7, 8, 19, 21, 27, 36, 43, 51, 61, 62, 63, 64, 65, 66, 67, 68	1, 2
Resting next root	+	+ +	+		Y	28	3, 4, 5	5, 6, 7, 8, 19, 21, 27, 36, 43, 51, 61, 62, 63, 64, 65, 66, 67, 68	1, 2
Resting on stone	+ + +	+ + +	+ + +	+ + +	Y	29	3, 4, 5	5, 6, 7, 8, 19, 21, 27, 36, 43, 51, 61, 62, 63, 64, 65, 66, 67, 68	5
Resting under crag			+ +		Y	30	3, 4, 5	5, 6, 7, 8, 19, 21, 27, 36, 43, 51, 61, 62, 63, 64, 65, 66, 67, 68	5, 6
Huddling	+	+ +	+ +	+	Y	31	3, 4, 5	27, 15, 28, 29, 30	15, 16, 17, 18, 19, 20, 21
Shivering	+	+			W, Sp	32	3, 4, 5	69	1, 2, 3, 4, 5, 6, 7
**4.Pairing behavior**									
Pairing grooming	+ + +	+ + +	+ + +		Y	33	3, 4, 5, 14	12, 17, 43, 44, 45, 47, 48, 61, 62, 63, 66, 68	19, 21
Kissing back	+ + +		+ +		Y	34	3, 4, 5, 14	8, 11, 53, 54	15, 16, 19, 21
Solicitation		+			Y	35	4, 9	1, 5, 6, 7, 30, 64, 68, 76	15, 16, 19, 21
**5．Mating behavior**									
Mounting	+ +	+ +			Y	36	1, 4, 9	39, 42, 54, 72, 80	15, 16, 19, 21
Copulation	+ +	+ +			Y	37	4, 9, 13, 14	1, 5, 6, 7, 30, 39, 42, 54, 72, 82, 83, 84, 85, 86	15, 16, 19, 21
Disturbing			+	+	Y	38	13	25, 30, 35, 43, 58	15, 16, 19, 21
Copulation call		+			Y	39	13	23	15, 16, 19, 21
**6．Parental behavior**									
Examination		+	+		Y	40	1, 3	8, 11, 30, 40, 41, 43, 45, 51	16, 19, 20
Holding tail		+			Y	41	1, 3	7, 30, 42, 63	16, 19, 20
Nursing		+ + +			Sp, S, A	42	1, 3, 5, 10, 14, 16	51, 55	16, 20
Sucking				+ +	Sp, S, A	43	3, 4, 8	13, 14, 15, 17, 18, 20, 42, 51	17
Nipple holding			+ +	+ + +	Y	44	3, 4, 8	13	17

Behaviors	Adult male	Adult female	Juvenile	Infant	Season	Order code	Posture	Act	Environment
Refusing to nurse		++			Y	45	1, 3, 5, 14	39, 49	16
Embracing	+	+++	+		Y	46	1, 2, 3, 10	41, 51	16, 19, 20
Kidnapping		+	+++		Y	47	1, 2, 3, 10	15, 23, 41, 42, 46	16, 17, 21
Kissing		++			Y	48	14	11	16, 19, 20
Carriaging		+++	+		Y	49	1, 10, 11, 12	41, 42	16, 17
Tantrumming			+	+++	Y	50	1, 3, 4, 5, 10, 11, 12, 14, 15, 16	1, 24, 25	17, 18
Allomother behavior		+	+++		S, A	51	14, 16	41, 43, 51	16, 21
**7. Superior behavior**									
Opening mouth	+	+	+++		Y	52	1, 2, 3, 8, 10, 11, 12	15, 16	15
Displaying	+++	+			Y	53	11, 12	25, 26, 35, 50, 58, 59	1, 2, 15
Supplementing	+++				Y	54	1, 2, 3, 4, 5, 8, 10, 11	1, 53, 54	15, 16, 18, 19, 20
Robbing food		++	+++		Y	55	1, 2, 3, 10, 11, 12	42, 47, 48	1, 2, 3, 15
**8. Threatening behavior**									
Staring	+	+	+		Y	56	1, 2, 3, 4, 10, 11	1, 29, 30, 56	15, 16, 18, 19, 20, 21
Staring each other	++				Y	57	1, 2, 3, 4, 10, 11	1, 29, 30, 56	15, 16, 18, 19, 20, 21
Hitting on the ground	++				Y	58	1	1, 4, 37, 46	15, 16, 18, 19, 20, 21
Baring teeth	++	+	+++		Y	59	1, 2, 3, 4, 10, 11	4, 16, 30, 53, 77	15, 16, 18, 19, 20, 21
Howling	++	+	+		Y	60	10, 11, 12	25, 30	16
Striding toward	+++				Y	61	10	30, 55	15
**9. Alert behavior**									

Behaviors	Adult male	Adult female	Juvenile	Infant	Season	Order code	Posture	Act	Environment
Vigilancing	+	+	+		Y	62	1, 2, 3, 10, 11, 12	2, 3, 30	15
LookIng around	+	+ +	+		Y	63	1, 2, 3, 10, 11, 12	2, 5, 6, 30	15
Roaring for alert	+	+			Y	64	1, 3, 10	15, 25, 29, 77	15, 16
Shaking branches for alert	+				Y	65	11, 12	25, 37, 50, 51, 58, 59	15
**10. Aggressive behavior**									
Driving away	+ +	+ +	+		Y	66	1, 3, 10, 11, 12	55	20
Lunging	+ +		+		Y	67	11	58, 59	15
Chasing	+ + +		+		Y	68	11	58, 59	15, 16, 18
Dashing	+	+ +			Y	69	10, 11, 12	58, 59	15
Grasping and hitting	+ +	+	+ +		Y	70	1, 2, 11, 12	37, 42, 54, 64, 68	15, 16, 18, 21
Wrestling			+ + +	+	Y	71	2	42, 45, 49	15, 16, 20, 21
Biting	+	+	+ +		Y	72	11, 12	8, 13, 15, 39	15, 16, 19, 20, 21
**11. Submissive behavior**									
Avoiding	+ +	+ + +	+		Y	73	1, 3, 4, 5, 10, 11	5, 6, 8, 16, 19, 73, 74	15, 16, 21
Surrendering	+	+ +	+ +		Y	74	3, 4, 9	36, 64, 72, 77	15, 16, 18, 19, 20, 21
Retreating	+		+		Y	75	3, 4	30, 53, 56, 73, 74	15, 16, 21
Crouching	+ + +	+ + +	+ + +		Y	76	6	19, 26	15, 16, 21
Fleeing	+ +		+ + +		Y	77	10, 11	57, 58, 59, 73, 74	15, 16, 21
**12. Friendly behavior**									
Waiting	+				Y	78	1, 2, 3, 10	4, 30, 53, 54, 76	15, 16
Opposite sitting		+ + +	+ + +		Y	79	3	8	15, 16
Backresting	+ + +	+ + +	+ +	+	Y	80	3	8	15, 16

Behaviors	Adult male	Adult female	Juvenile	Infant	Season	Order code	Posture	Act	Environment
Holding hand		++	+		Y	81	1, 3, 4, 5, 10, 11	46	15, 16
Lightly patting	+	+	+++	+++	Y	82	1, 3, 5	37	15, 16
Sighting exchange	++	+++	+++	+	Y	83	1, 2, 3, 4	30, 35, 73, 74	15, 16
Playing	+	+	+++	++	Y	84	1, 3, 4, 5, 10, 11, 12	1, 5, 6, 37, 44, 46, 49	17, 18, 19, 21
**13. Affinitive behavior**									
Approaching	+++	++	++		Y	85	10, 11	55, 73, 74	15, 16, 19, 20, 21
Following	+++	++	+	++	Y	86	10, 11	58, 59, 73, 74	15, 16, 19, 20, 21
Leaving	+++	+++	++	+++	Y	87	10, 11	57, 73, 74	15, 16, 19, 20, 21
Contact sitting	++	++	+		Y	88	3	57, 73, 74	15, 16, 19, 20, 21
Embracing		+	++		Y	89	2, 3	51	15, 16, 19, 20, 21
Huddling	+	+	++	+	Y	90	2, 3	51, 54	15, 16, 19, 20, 21
Holding lumbar	+	+		+++	Y	91	2, 3	51, 70, 88	15, 16, 19, 20, 21
Caressing		++	+++		Y	92	1, 3, 4, 5	37, 39	15, 16, 19, 20, 21
Allogrooming	++	+++	++		Y	93	1, 2, 3, 4, 5	43	15, 16, 19, 20, 21
Hipping present		+++	++		Y	94	1	5, 6, 7, 53, 75, 76	15, 16, 18
Showing a penis		+++	+		Y	95	1	5, 6, 7, 8, 65	15, 18, 21
Non-sexual mounting			++		Y	96	1, 9, 10	53, 75, 80, 84, 86	20, 21
Kissing	+++	++	+		Y	97	1, 3, 4, 5	11, 12, 33, 34	21

Behaviors	Adult male	Adult female	Juvenile	Infant	Season	Order code	Posture	Act	Environment
**14. Aggregation behavior**									
Matrilineal grouping		+ + +	+ +	+ + +	Y	98	1, 2, 3, 4, 5, 8, 10		20
Male grouping	+ + +		+ +		Y	99	1, 2, 3, 4, 5, 8, 10		15
Female grouping		+ + +	+		Y	100	1, 2, 3, 4, 5, 8, 10		16, 17, 18
Immature grouping				+ +	Y	101	1, 2, 3, 4, 5, 8, 10		17, 18
Single	+ + +	+	+ +		Y	102	1, 2, 3, 4, 5, 8, 10		22
Lonely	+ +				W, Sp	103	1, 2, 3, 4, 5, 8, 10		15, 16, 22
**15. Resting behavior**									
Standing and sinking waist	+	+	+ +		Y	104	1	21, 53, 72	3, 4, 5, 6
Standing and unfolding waist	+ + +	+	+ +		Y	105	1	21, 53, 70	3, 4, 5, 6
Sitting and crouching	+ + +	+ + +	+ + +	+ + +	Y	106	3, 6	21, 66	1, 2, 3, 4, 5, 6
Sitting and unfolding legs	+ + +	+ +	+ + +		Y	107	3	21, 64, 68	1, 2, 3, 4, 5, 6
Groveling and crouching	+ +		+		Y	108	4, 6	21, 62, 66	2, 3, 4, 5, 6
Groveling and unfolding legs	+ +	+	+ +		Y	109	4	21, 64, 68	1, 2, 3, 4, 5, 6
Lying and unfolding legs	+ +	+	+ +		Y	110	5	21, 64, 68	1, 2, 3, 4, 5, 6
Yawning	+	+	+		Y	111	1, 2, 3, 4, 5, 10, 11	21, 31, 32	15, 16, 17, 18
**16. Locomotive behavior**									
Walking	+ + +	+ + +	+ + +	+ +	Y	112	10	55, 56	2, 3, 4, 5, 6, 7
Walking on three legs	+ +	+ +	+		Y	113	10	55, 56	7
Walking on hindlegs	+ + +	+ + +	+ + +	+	Y	114	10	54, 55, 56	1, 2, 3, 4, 5, 6, 7
Trotting	+	+ +	+ +	+ + +	Y	115	11	58	2, 3, 4, 5, 6, 7

Behaviors	Adult male	Adult female	Juvenile	Infant	Season	Order code	Posture	Act	Environment
Trotting on three legs	+				W	116	11	58	7
Galloping	+ + +	+ +	+ + +	+ +	Y	117	11	58	2, 3, 4, 5, 6, 7
Walking forward	+ + +	+ + +	+ + +	+ + +	Y	118	10	55	1, 2, 3, 4, 5, 6, 7
Walking aside	+ +	+ +	+ +	+ +	Y	119	10	57	2, 3, 4, 5, 6, 7
Jumping	+ +	+ + +	+ + +	+ +	Y	120	12	59	1, 2, 3, 4, 5, 6, 7
Hanging	+ +	+	+ + +		Y	121	8	42, 50	1, 2
Swing			+ +	+ + +	Y	122	12	42, 50	1, 2
Climbing tree	+	+ +	+ +	+	Y	123	7, 11, 12	40, 42, 54, 58, 59	1, 2
Going to the field	+ +	+ +	+	+	Y	124	3, 4, 5, 7, 10, 11, 12	53, 55	1, 2
**17.Cleaning behavior**									
Wipping hands	+ +		+		Y	125	1, 3	8, 12, 17, 61	15, 16
Picking feet			+		Y	126	3	8, 38, 44	15, 16
Cleanning the wound	+				Sp, A	127	3	8, 12, 15, 17, 45	15, 16
Shaking fur	+ +	+ +	+		Y	128	1, 10	2, 53, 55, 69	15, 16, 18
Self-grooming	+	+ +	+ +		Y	129	3	8, 43	15, 16, 18
**18． Miscellaneous behavior**									
Switching tail	+	+ + +	+ +	+ + +	Y	130	1, 4	76	16, 22
Raising tail	+ + +	+ + +	+ + +	+ +	Y	131	1, 4	75	15, 16, 19, 20, 21, 22
Curious			+	+	Sp, S, A	132	1, 2, 3	1, 30	17, 18
Fiddlling			+ +	+	Y	133	1, 2, 3	1, 30, 45	17, 18
Itching	+ +	+ +	+ + +	+	Y	134	1, 3, 4, 5	36, 42, 44, 65	15, 16
Sucking finger	+	+	+ +		Y	135	1, 3, 4, 10	8, 11, 15, 17, 61	15, 16, 17, 18

[1] '+' denotes the relative frequency tier for a given age–sex group. With '+++' representing the top tertile (highest 33.3%), '++' the middle tertile (33.3%), and '+' the bottom tertile (lowest 33.3%) of the observed occurrence frequency distribution, while blank cells indicate the behavior was not observed for that class.
